# Leveraging Homologous Hypotheses for Increased Efficiency in Tumor Growth Curve Testing

**DOI:** 10.21203/rs.3.rs-3242375/v1

**Published:** 2023-08-17

**Authors:** Alan D. Hutson, Han Yu, Kristopher Attwood

**Affiliations:** 1 Roswell Park Comprehensive Cancer Center, Department of Biostatistics and Bioinformatics, Elm and Carlton Streets, Buffalo, NY 14623.

## Abstract

In this note, we present an innovative approach called ”homologous hypothesis tests” that focuses on cross-sectional comparisons of average tumor volumes at different time-points. By leveraging the correlation structure between time-points, our method enables highly efficient per time-point comparisons, providing inferences that are highly efficient as compared to those obtained from a standard two-sample *t*-test. The key advantage of this approach lies in its user-friendliness and accessibility, as it can be easily employed by the broader scientific community through standard statistical software packages.

## 1 Introduction

Tumor growth modeling in pre-clinical cancer research is a pivotal analysis that has been extensively explored in countless research papers. However, the utilization of animal models in this context can impose significant financial burdens due to their high costs. Consequently, optimizing testing procedures for growth curve modeling becomes crucial to ensure cost-efficiency without compromising accuracy and reliability.

**Figure 1:**
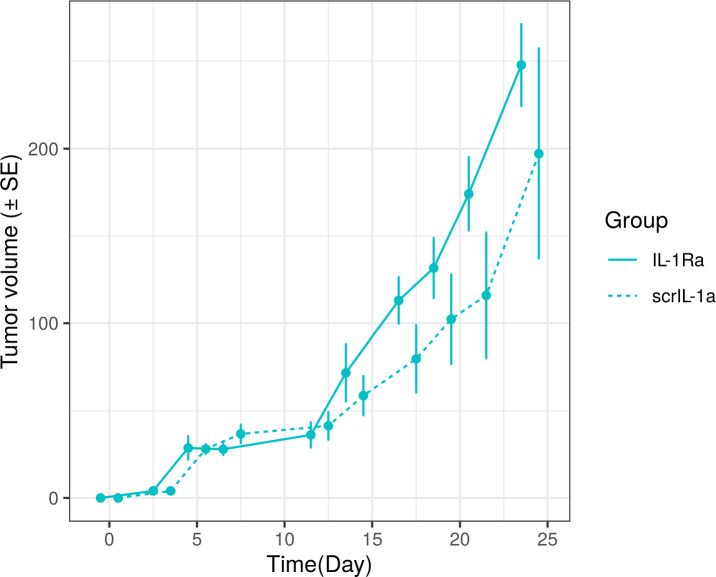
Example Tumor Growth Curve.

In terms of background, and without loss of generality, let us focus on a two group comparison of an experimental treatment A versus treatment B in terms of comparing changes in tumor volume over time. Figure 1 depicts a commonly encountered plot in the literature, showcasing the average tumor volume growth (*mm*^3^) for mice treated with IL-1Ra versus scrIL-1a. plotted against time[1]. The scrIL-1a values are offset for easier readability.

One strategy for modeling rates of change in tumor volume generally assume that the log tumor volume has linear relationship with time [2], with time measured on a continuous scale. Zavrakidis *et al*.[2] recommend a linear regression model with an autoregressive (AR-1) covariance structure for analyzing log-transformed tumor volumes. This model effectively accounts for the correlation among repeated measurements per mouse and provides unbiased results in comparing tumor growth rates between treatment groups. However, the accuracy of the model’s performance depends on the correct specification of the variance-covariance structure, as misspecification can affect the type I error and coverage rates. A similar study was carried forth in patient derived xenograft models[3].

A series of nonlinear mixed-effects models that mathematically describe tumor size dynamics in cancer patients undergoing anticancer drug treatment has been developed as the Drug Disease Model Resources (DDMoRe) repository for oncology models[4]. More recently, Forrest *et al*.[5] propose a nonparametric approach to overcome the linearity assumptions using regression splines in a generalized additive mixed model to estimate group-level response trends in logarithmically scaled tumor volume. This approach improves the fidelity of describing nonlinear growth scenarios and enhances statistical power for detecting differences between treatment regimens. Vaghi *et al*.[6] analyzed tumor growth kinetics using a nonlinear mixed-effects approach and found that the Gompertz model provided the best fit to the experimental data. They confirmed a correlation between the Gompertz model parameters and proposed a reduced Gompertz function that improved predictive accuracy and precision, offering potential clinical applications in personalized tumor age prediction based on limited diagnostic data.

Alternatively, when monitoring tumor volume at specific time intervals, a useful approach is the application of a standard mixed model analysis of variance. This method treats each time-point as a distinct category and incorporates factors such as treatment, time, and their interaction, providing a nonparametric perspective on the relationship between time and tumor volume. If a significant overall difference in growth curves is observed, the subsequent step involves examining cross-sectional comparisons at each time point as specific contrasts within the mixed model. In general, these contrasts are simplified to two-sample *t*-tests assuming normality, disregarding the correlation structure between time points. The main objective of these cross-sectional analyses is to statistically determine the time point at which the growth curve diverges and ascertain whether the growth curves remain separated in subsequent measurements. This information proves valuable in understanding the temporal dynamics of tumor growth and treatment effects. This analytical approach can be argued to be the predominant analysis presented in the field of tumor volume growth.

In Section 2, we provide a precise definition of the homologous hypothesis and draw a clear contrast between this approach and the current mean-based tests. Section 3 delves into formulating the homologous hypothesis within the regression framework, leading to a more concise presentation of the results. Additionally, in Section 4, we offer power comparisons between the homologous hypothesis test and the traditional two-sample *t*-test, demonstrating the robustness and effectiveness of our method. To illustrate the practicality of our approach, we provide a real-life example in Section 5, followed by concluding remarks in the final section. Our intention is to make this methodology accessible and applicable, fostering advancements in tumor volume analysis and facilitating broader adoption within the scientific community.

## 2 Definition of a Homologous Hypothesis

Let $Y_{x_{i},ij}$ denote the tumor volume for the *i*th animal, i=1,2,⋯,n, at the *j*th time-point, j=1,2,⋯,m, and let $x_{i}$ indicate the treatment assignment for the *i*th animal ($x_{i}=0$ for treatment A, $x_{i}=1$ for treatment B), with the total sample size denoted as $n=n_{0}+n_{1}$. In cross-sectional analyses the null hypothesis of interest is to compare the mean tumor volume between treatment A and treatment B at specific time, given as

(2.1) $$H_{0}:E\left(Y_{0,j}\right)=E\left(Y_{1,j}\right),$$

where $E\left(Y_{0,j}\right)$ and $E\left(Y_{1,j}\right)$ are the expected values for tumor volumes for treatment A and treatment B at time j. The alternative hypothesis may be two-sided or one-sided depending upon the needs of the analyst. This test is generally carried out on the raw values or log-transformed tumor volume values using a two-sample *t*-test.

Now, let us assume a linear relationship between between the mean tumor volume at time point j and time-point j−1 for treatment groups A and B, respectively, and given as follows:

(2.2) $$E\left(Y_{0,j}|Y_{0,j-1}=y_{0,j-1}\right)=E\left(Y_{0,j}\right)+\rho_{0,j}\frac{\sigma_{Y_{0,j}}}{\sigma_{Y_{0,j-1}}}(y_{0,j-1}-E\left(Y_{0,j-1}\right)),$$

(2.3) $$E\left(Y_{1,j}|Y_{1,j-1}=y_{1,j-1}\right)=E\left(Y_{1,j}\right)+\rho_{1,j}\frac{\sigma_{Y_{1,j}}}{\sigma_{Y_{1,j-1}}}(y_{1,j-1}-E\left(Y_{1,j-1}\right)),$$

where $\rho_{0,j}$ is the correlation between $Y_{0,j}$ and $Y_{0,j-1},\;\rho_{1,j}$ is the correlation between $Y_{1,j}$ and $Y_{1,j-1},\;\sigma_{Y_{0,j}}$ and $\sigma_{Y_{0,j-1}}$ are the standard deviations for $Y_{0,j}$ and $Y_{0,j-1}$, respectively, and $\sigma_{Y_{1,j}}$ and $\sigma_{Y_{1,j-1}}$ are the standard deviations for $Y_{1,j}$ and $Y_{1,j-1}$, respectively.

An immediate examination of equations (2.2) and (2.3) reveals that $E\left[Y_{0,j}|Y_{0,j-1}=\right.\left.E\left(Y_{0,j-1}\right)\right]=E\left(Y_{0,j}\right)$ and $E\left[Y_{1,j}|Y_{1,j-1}=E\left(Y_{1,j-1}\right)\right]=E\left(Y_{1,j}\right)$. This interesting relationship suggests a potentially more efficient approach for testing (2.1), leveraging the correlation between $Y_{0,j}$ and $Y_{0,j-1}$, as well as $Y_{1,j}$ and $Y_{1,j-1}$. Furthermore, it is worth noting that $E\left(Y_{0,j}|Y_{0,j-1}=y_{0,j-1}\right)=E\left(Y_{0,j}\right)$ holds true when $\rho_{0,j}=0$, and similarly, $E\left(Y_{1,j}|Y_{1,j-1}=y_{1,j-1}\right)=E\left(Y_{1,j}\right)$ when $\rho_{1,j}=0$. In other words, no additional information is gained in cases where there is no correlation between time points. However, in general, tumor growth curve models exhibit a high degree of correlation between adjacent time points. The sets of dependence relationships between tumor volumes over time form the basis for our concept of a *homologous hypothesis* as an alternative to the standard cross-sectional hypothesis at (2.1) for comparing two means.

### Definition of a Homologous Hypothesis.

We define the homologous null hypothesis for time point j, j>1, as follows:

(2.4) $$H_{0}\;:E\left(Y_{0,j}|Y_{0,j-1}={\overset{‾}{y}}_{0,j-1}\right)=E\left(Y_{1,j}|Y_{1,j-1}={\overset{‾}{y}}_{1,j-1}\right),$$

where ${\overset{‾}{y}}_{0,j-1}=\sum_{i=1}^{n}.Y_{x_{i},ij-1}\left(1-x_{i}\right)/n_{0}$ and ${\overset{‾}{y}}_{1,j-1}=\sum_{i=1}^{n}.Y_{x_{i},ij-1}x_{i}/n_{1}$ are the moment estimators for the expected tumor volumes $E\left(Y_{0,j-1}\right)$ and $E\left(Y_{1,j-1}\right)$, respectively, at time-point j−1.

The similarity between the standard cross-sectional null hypothesis at (2.1) and the homologous null hypothesis at (2.4) may be seen by noting that

(2.5) $$E\left(Y_{0,j}|Y_{0,j-1}={\overset{‾}{y}}_{0,j-1}\right)=E\left(Y_{0,j}\right)+\rho_{0,j}\frac{\sigma_{Y_{0,j}}}{\sigma_{Y_{0,j-1}}}({\overset{‾}{y}}_{0,j-1}-E\left(Y_{0,j-1}\right)),$$

(2.6) $$E\left(Y_{1,j}|Y_{1,j-1}={\overset{‾}{y}}_{1,j-1}\right)=E\left(Y_{1,j}\right)+\rho_{1,j}\frac{\sigma_{Y_{1,j}}}{\sigma_{Y_{1,j-1}}}({\overset{‾}{y}}_{1,j-1}-E\left(Y_{1,j-1}\right)),.$$

i.e., $E\left(Y_{0,j}|Y_{0,j-1}{\overset{‾}{y}}_{0,j-1}\right)$ is within a neighborhood of $E\left(Y_{0,j}\right)$ and $E\left(Y_{1,j}|Y_{0,j-1}{\overset{‾}{y}}_{1,j-1}\right)$ is within a neighborhood of $E\left(Y_{1,j}\right)$.

In particular, through standard central limit arguments with bounded variances assumed, we have ${\overset{‾}{y}}_{0,j-1}\overset{p}{\to }E\left(Y_{0,j-1}\right)$ and ${\overset{‾}{y}}_{1,j-1}\overset{p}{\to }E\left(Y_{1,j-1}\right)$ as n→∞. Therefore, in an asymptotic sense, the homologous hypothesis stated in (2.4) can be considered equivalent to the standard cross-sectional hypothesis in (2.1). In other words we can rewrite the homologous null hypothesis at (2.4) as

(2.7) $$H_{0}\;:E\left(Y_{0,j}\right)-E\left(Y_{1,j}\right)=o_{p}\left(1\right).$$

The standard cross-sectional null hypothesis at (2.1) and the homologous null hypothesis at (2.4) exhibit subtle differences, except when $\rho_{0,j}=0$ and $\rho_{1,j}=0$. However, the primary reason for rejecting the homologous null hypothesis lies in the discrepancies between the population mean growth tumor volumes $E\left(Y_{1,j}\right)-E\left(Y_{0,j}\right)$ at time j. Emphasizing this point, if the investigator is willing to accept these subtle distinctions between the standard cross-sectional null hypothesis and the homologous null hypothesis substantial gains in statistical efficiency may be achieved. This can be accomplished by capitalizing on the correlation structure between successive tumor growth values over time. This in turn can reduce sample size requirements dramatically, where certain animal models may cost several thousand dollars per unit.

Furthermore, if the parameters for equations (2.2) and (2.3) are estimated via standard least-squares regression of $Y_{0,j}$ on $Y_{0,j-1}$ and $Y_{1,j}$ on $Y_{1,j-1}$ we arrive at the following estimators:

(2.8) $$\overset{ˆ}{E}\left(Y_{0,j}|Y_{0,j-1}=y_{0,j-1}\right)={\overset{‾}{y}}_{0,j}+{\overset{ˆ}{\rho }}_{0,j}\frac{{\overset{ˆ}{\sigma }}_{Y_{0,j}}}{{\overset{ˆ}{\sigma }}_{Y_{0,j-1}}}\left(y_{0,j-1}-{\overset{‾}{y}}_{0,j-1}\right),$$

(2.9) $$\overset{ˆ}{E}\left(Y_{1,j}|Y_{1,j-1}=y_{1,j-1}\right)={\overset{‾}{y}}_{1,j}+{\overset{ˆ}{\rho }}_{1,j}\frac{{\overset{ˆ}{\sigma }}_{Y_{1,j}}}{{\overset{ˆ}{\sigma }}_{Y_{1,j-1}}}\left(y_{1,j-1}-{\overset{‾}{y}}_{1,j-1}\right),$$

where ${\overset{‾}{y}}_{0,j-1}$ and ${\overset{‾}{y}}_{1,j-1}$ are defined above,

$${\overset{‾}{y}}_{0,j}=\overset{ˆ}{E}\left(Y_{0,j}\right)=\frac{\sum_{i=1}^{n}\;Y_{x_{i},ij}\left(1-x_{i}\right)}{n_{0}}\;\text{and}\;{\overset{‾}{y}}_{1,j}=\overset{ˆ}{E}\left(Y_{1,j}\right)=\frac{\sum_{i=1}^{n}\;Y_{x_{i},ij}x_{i}}{n_{1}}$$

$${\overset{ˆ}{\sigma }}_{Y_{0,j}}^{2}=\frac{\sum_{i=1}^{n}\;\left(1-x_{i}\right){\left(Y_{x_{i},ij}-{\overset{‾}{y}}_{0,j}\right)}^{2}}{n_{0}-1}\;\text{and}\;{\overset{ˆ}{\sigma }}_{Y_{0,j-1}}^{2}=\frac{\sum_{i=1}^{n}\;\left(1-x_{i}\right){\left(Y_{x_{i},ij-1}-{\overset{‾}{y}}_{0,j-1}\right)}^{2}}{n_{0}-1},$$

$${\overset{ˆ}{\sigma }}_{Y_{1,j}}^{2}=\frac{\sum_{i=1}^{n}\;x_{i}{\left(Y_{x_{i},ij}-{\overset{‾}{y}}_{1,j}\right)}^{2}}{n_{1}-1}\;\text{and}\;{\overset{ˆ}{\sigma }}_{Y_{1,j-1}}^{2}=\frac{\sum_{i=1}^{n}\;x_{i}{\left(Y_{x_{i},ij-1}-{\overset{‾}{y}}_{1,j-1}\right)}^{2}}{n_{1}-1},$$

$${\overset{ˆ}{\rho }}_{0,j}=\frac{\sum_{i=1}^{n}\;\left(1-x_{i}\right)\left(Y_{x_{i},ij}-{\overset{‾}{y}}_{0,j}\right)\left(Y_{x_{i},ij-1}-{\overset{‾}{y}}_{0,j-1}\right)}{\sqrt{\sum_{i=1}^{n}\;\left(1-x_{i}\right){\left(Y_{x_{i},ij}-{\overset{‾}{y}}_{0,j}\right)}^{2}}\sqrt{\sum_{i=1}^{n}\;\left(1-x_{i}\right){\left(Y_{x_{i},ij-1}-{\overset{‾}{y}}_{0,j-1}\right)}^{2}}}$$

$${\overset{ˆ}{\rho }}_{1,j}=\frac{\sum_{i=1}^{n}\;x_{i}\left(Y_{x_{i},ij}-{\overset{‾}{y}}_{1,j}\right)\left(Y_{x_{i},ij-1}-{\overset{‾}{y}}_{1,j-1}\right)}{\sqrt{\sum_{i=1}^{n}\;x_{i}{\left(Y_{x_{i},ij}-{\overset{‾}{y}}_{1,j}\right)}^{2}}\sqrt{\sum_{i=1}^{n}\;x_{i}{\left(Y_{x_{i},ij-1}-{\overset{‾}{y}}_{1,j-1}\right)}^{2}}}.$$

Now, it should be clear from (2.8) and (2.9) that the sample estimators for the conditional and unconditional are identically the sample mean at time j, i.e.,

(2.10) $$\overset{ˆ}{E}\left(Y_{0,j}|Y_{0,j-1}={\overset{‾}{y}}_{0,j-1}\right)=\overset{ˆ}{E}\left(Y_{0,j}\right)={\overset{‾}{y}}_{0,j},$$

(2.11) $$\overset{ˆ}{E}\left(Y_{1,j}|Y_{1,j-1}={\overset{‾}{y}}_{1,j-1}\right)=\overset{ˆ}{E}\left(Y_{1,j}\right)={\overset{‾}{y}}_{1,j}.$$

However,

(2.12) $$\left.Var\left[Y_{0,j}|Y_{0,j-1}=y_{0,j-1}\right)\right]=\mathrm{Var}\left(Y_{0,j}\right)(1-\rho_{0,j}^{2}),$$

(2.13) $$\left.Var\left[Y_{1,j}|Y_{1,j-1}=y_{1,j-1}\right)\right]=\mathrm{Var}\left(Y_{1,j}\right)(1-\rho_{1,j}^{2}).$$

Thus if the correlation between tumor volumes at time point j and j−1 is strong a high degree of efficiency can be gained in terms of testing the homologous hypothesis stated at (2.4) as compared to the standard cross-sectional hypothesis in (2.1).

The variance estimates for $\overset{ˆ}{E}\left(Y_{0,j}|Y_{0,j-1}={\overset{‾}{y}}_{0,j-1}\right)$ and $\overset{ˆ}{E}\left(Y_{1,j}|Y_{1,j-1}={\overset{‾}{y}}_{1,j-1}\right)$ at (2.10) and (2.11), respectively, follow from standard least-squares theory and are as follows:

(2.14) $$\hat{Var}(\overset{ˆ}{E}(Y_{0,j}|Y_{0,j-1}={\overset{‾}{y}}_{0,j-1}))=\frac{MSE_{0}}{n_{0}},$$

(2.15) $$\hat{Var}(\overset{ˆ}{E}(Y_{1,j}|Y_{1,j-1}={\overset{‾}{y}}_{1,j-1}))=\frac{MSE_{1}}{n_{0}},$$

Where

(2.17) $$MSE_{0}=\frac{\sum_{i}^{n_{0}}\;{\left[Y_{0,ij\;}-\;\overset{ˆ}{E}\left(Y_{0,j}|Y_{0,j-1}=y_{0,ij-1}\right)\right]}^{2}}{n_{0}-2},$$

(2.18) $$MSE_{1}=\frac{\sum_{i}^{n_{1}}\;{\left[Y_{1,ij}\;-\;\overset{ˆ}{E}\left(Y_{1,j}|Y_{1,j-1}=y_{1,ij-1}\right)\right]}^{2}}{n_{1}-2},$$

and $\overset{ˆ}{E}\left(Y_{0,j}|Y_{0,j-1}=y_{0,j-1}\right)$ and $\overset{ˆ}{E}\left(Y_{1,j}|Y_{1,j-1}=y_{1,j-1}\right)$ are given at (2.8) and (2.9), respectively.

## 3 Regression Framework for Testing a Homologous Hypothesis

We can create a more streamlined approach for testing the homologous hypothesis

(3.1) $$H_{0}\;:E\left(Y_{0,j}|Y_{0,j-1}={\overset{‾}{y}}_{0,j-1}\right)=E\left(Y_{1,j}|Y_{1,j-1}={\overset{‾}{y}}_{1,j-1}\right),$$

at time point j, j>1, using a regression framework. Combining equations (2.5) and (2.6) into a single regression framework at time j we arrive at the model

(3.2) $$Y_{x_{i},ij}=\beta_{0,j}+\beta_{1,j}x_{i}+\beta_{2,j}y_{x_{i},j-1}+\beta_{3,j}x_{i}y_{x_{i},j-1}+\epsilon_{ij},i=1,\;2,\cdots ,n,$$

where as before $Y_{x_{i},ij}$ denotes the tumor volume for the *i*th animal, i=1,2,⋯,n, at the *j*th time-point, $j=1,\;2,\cdots ,m,\;x_{i}$ indicates the treatment assignment for the *i*th animal ($x_{i}=0$ for treatment A, $x_{i}=1$ for treatment B) and the $\epsilon_{i}$’s are assumed independent and identically distributed (i.i.d.), $\epsilon_{i}\sim N(0,\sigma_{j}^{2})$. As will be evident from the discussion below, the regression framework provides a streamlined approach for estimating the standard errors of our quantities of interest and utilizing the well-known classical inferential framework.

Defining the regression model at (3.2) allows us to reformulate our conditional estimators $\overset{ˆ}{E}\left(Y_{0,j}|Y_{0,j-1}={\overset{‾}{y}}_{0,j-1}\right)$ at (2.8) and $\overset{ˆ}{E}\left(Y_{1,j}|Y_{1,j-1}={\overset{‾}{y}}_{1,j-1}\right)$ at (2.9) as follows:

(3.3) $$\overset{ˆ}{E}\left(Y_{0,j}|Y_{0,j-1}={\overset{‾}{y}}_{0,j-1}\right)={\overset{ˆ}{\beta }}_{0,j}+{\overset{ˆ}{\beta }}_{2,j}{\overset{‾}{y}}_{0,j-1}={\overset{‾}{y}}_{0,j},$$

(3.4) $$\overset{ˆ}{E}\left(Y_{1,j}|Y_{1,j-1}={\overset{‾}{y}}_{1,j-1}\right)={\overset{ˆ}{\beta }}_{0,j}+{\overset{ˆ}{\beta }}_{1,j}+({\overset{ˆ}{\beta }}_{2,j}+{\overset{ˆ}{\beta }}_{3,j}){\overset{‾}{y}}_{1,j-1}={\overset{‾}{y}}_{1,j},$$

where ${\overset{ˆ}{\beta }}_{0,j}$, ${\overset{ˆ}{\beta }}_{1,j}$, ${\overset{ˆ}{\beta }}_{2,j}$, and ${\overset{ˆ}{\beta }}_{3,j}$ are standard least-squares regression slope estimators. This approach leads to the mean difference estimator

(3.5) $${\overset{ˆ}{D}}_{j}\;=\overset{ˆ}{E}\left(Y_{1,j}|Y_{1,j-1}={\overset{‾}{y}}_{1,j-1}\right)={\overset{‾}{y}}_{1,j-1})-\overset{ˆ}{E}\left(Y_{0,j}|Y_{0,j-1}={\overset{‾}{y}}_{0,j-1}\right)\;\;={\overset{ˆ}{\beta }}_{1,j}+{\overset{ˆ}{\beta }}_{2,j}\left({\overset{‾}{y}}_{1,j-1}-{\overset{‾}{y}}_{0,j-1}\right)+{\overset{ˆ}{\beta }}_{3,j}{\overset{‾}{y}}_{1,j-1}.\;\;={\overset{‾}{y}}_{1,j}-{\overset{‾}{y}}_{0,j}$$

In terms of matrix formulation let

(3.6) $$Y_{j}=\left[\begin{array}{c} Y_{0,1j}\\ Y_{0,2j}\\ \vdots \\ Y_{0,n_{0}j}\\ Y_{1,n_{0}+1j}\\ Y_{1,n_{0}+2j}\\ \vdots \\ Y_{1,n_{0}+n_{1}j} \end{array}\right]\;X_{j}=\left[\begin{array}{cccc} 1 & 0 & y_{0,1j-1} & 0\\ 1 & 0 & y_{0,2j-1} & 0\\ \vdots & \vdots & & \\ 1 & 0 & y_{0,n_{0}j-1} & 0\\ 1 & 1 & y_{1,n_{0}+1j-1} & y_{1,n_{0}+1j-1}\\ 1 & 1 & y_{1,n_{0}+2j-1} & y_{1,n_{0}+2j-1}\\ \vdots & \vdots & & \\ 1 & 1 & y_{1,n_{0}+n_{1j-1}} & y_{1,n_{0}+n_{1}j-1} \end{array}\right].$$

The regression model at (3.2) may be re-written compactly in matrix form as

(3.7) $$Y_{j}=X_{j}\beta_{j}+\epsilon_{j},$$

where the vector of $\epsilon_{ij}$’s are i.i.d. $N(0,\sigma_{j}^{2})$ and $Y_{j}$ and $X_{j}$ are given at (3.6).

It follows from standard linear model least-squares theory that the 4 × 1 vector of regression coefficient estimators has the following form:

(3.8) $${\overset{ˆ}{\beta }}_{j}={\left(X_{j}^{\prime }X_{j}\right)}^{-1}X_{j}^{\prime }Y_{j}.$$

To obtain the regression form of the estimator ${\overset{ˆ}{D}}_{j}=\overset{ˆ}{E}\left(Y_{0,j}|Y_{0,j-1}={\overset{‾}{y}}_{0,j-1}\right)={\overset{‾}{y}}_{0,j-1})-\overset{ˆ}{E}\left(Y_{1,j}|Y_{1,j-1}={\overset{‾}{y}}_{1,j-1}\right)$ first define the 1 × 4 vector:

(3.9) $$z_{D_{j}}=\left(0,\;1,{\overset{‾}{y}}_{1,j-1}-{\overset{‾}{y}}_{0,j-1},{\overset{‾}{y}}_{1,j-1}\right).$$

Then

(3.10) $${\overset{ˆ}{D}}_{j}=z_{D}{\overset{ˆ}{\beta }}_{j}.$$

The sample variance of ${\overset{ˆ}{D}}_{j}$ (3.10) based on standard linear models formulations is given as:

(3.11) $$s_{{\overset{ˆ}{D}}_{j}}^{2}=z_{D_{j}}s^{2}({\overset{ˆ}{\beta }}_{j})z_{D_{j}}^{\prime },$$

where $SSE_{j}=Y_{j}^{\prime }Y_{j}-{\overset{ˆ}{\beta }}_{j}^{\prime }X_{j}^{\prime }Y_{j},\;MSE_{j}=SSE_{j}/(n-4)$, and $s^{2}({\overset{ˆ}{\beta }}_{j})=MSE_{j}{\left(X_{j}^{\prime }X_{j}\right)}^{-1}$.

Under model assumptions stated at (3.7) we have that

(3.12) $$\frac{{\overset{ˆ}{D}}_{j}\;-\;D}{s_{{\overset{ˆ}{D}}_{j}}}\sim t_{n-4},$$

where ${\overset{ˆ}{D}}_{j}$ is defined at (3.11) and $s_{{\overset{ˆ}{D}}_{j}}^{2}$ is defined at (3.11). The distributional result at (3.12) follows from standard least-squares theory. The homologous hypothesis test is available within the R homologous package available at GitHub (https://github.com/hyu-ub/homologous).

## 4 Simulation Study

We carried out a simulation study based on the regression model defined at (3.2). Without loss of generality we set $\beta_{0,j}=0$. Tables 2–4 contain simulated power results for the homologous hypothesis stated at (2.4) as compared to the standard cross-sectional hypothesis in (2.1) across various values of σ, $\beta_{1,j}$, $\beta_{2,j}$ and $\beta_{3,j}$ at α=0.05 with two-sided alternatives. Each simulation consisted 1,000 Monte Carlo replications the $y_{x_{i},j-1}$ ‘s were simulated for each replication using a standard N(0,1) distribution. In practical terms this simulation would be similar to working on the log-transformed tumor volumes. Note that when $\beta_{2,j}=0$ and $\beta_{3,j}=0$ the homologous hypothesis stated the standard cross-sectional hypothesis are equivalent with slight variations in the test due to different degress of freedom for the null *t*-distributions.

As previously mentioned, the homologous hypothesis (2.4) and the standard cross-sectional hypothesis (2.1) share similarities but are not entirely equivalent. To calibrate the simulation results, we establish the equality: $\rho_{0,j}\frac{\sigma_{Y_{0,j}}}{\sigma_{Y_{0,j-1}}}({\overset{‾}{y}}_{0,j-1}-E(Y_{0,j-1}))=\rho_{1,j}\frac{\sigma_{Y_{1,j}}}{\sigma_{Y_{1,j-1}}}({\overset{‾}{y}}_{1,j-1}-E(Y_{1,j-1}))$ within the homologous testing framework. Consequently, the primary factor influencing the power values is the disparity between Treatment A and Treatment B. By allowing the above equality to vary across replications, the power values for the homologous test would exhibit an increase.

The correlation between time point j−1 and time point j in log-transformed tumor volumes varies as σ changes from 0.4 to 1, with Table 2 showing the highest correlation and Table 4 the lowest. As expected, when $\beta_{2,j}=0$ and $\beta_{3,j}=0$, both tests yield nearly equivalent results. However, the power of the homologous test is significantly enhanced in cases of high correlation between time points, as demonstrated in Table 2 where σ=0.4, $\beta_{1,j}=1$, $\beta_{2,j}=1$, and $\beta_{3,j}=0$, resulting in a power of 0.968, compared to 0.386 for the standard two-sample *t*-test. Moreover, even with moderate correlation between time points, there are still considerable power gains. For example, in Table 2, when σ=1, $\beta_{1,j}=1$, $\beta_{2,j}=1$, and $\beta_{3,j}=0$, the power of the homologous test is 0.424, compared to 0.282 for the standard two-sample *t*-test. These findings highlight the significance of considering correlation between time points when conducting tests, as it can lead to substantial improvements in statistical power.

## 5 Example

**Figure 2:**
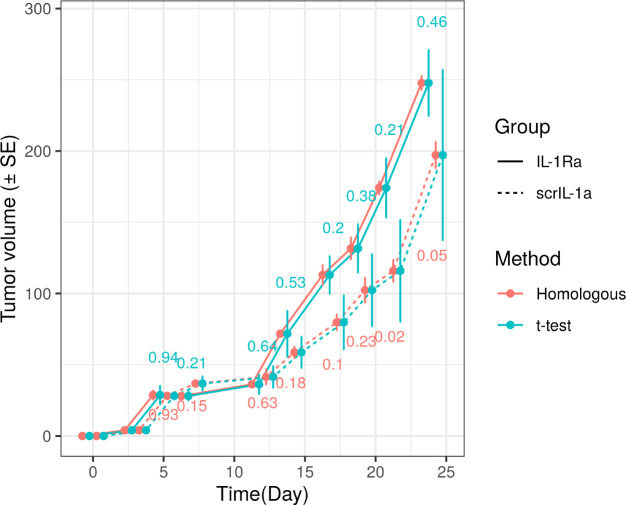
Example Homologous Tumor Growth Curve. The error bars are standard errors (SEs) estimated using two methods. The p-values for comparing mean tumor volumes at each time point are shown.

To demonstrate our method, we analyzed the tumor growth curves from the study by Sass et al[1], which showed that the IL-1*α* expression facilitate tumor cell proliferation. The data showed that IL-1*α* knockdown by shIL-1*α* can delay the tumor growth when compared with the control group (scrIL-1*α*). In addition, the blockade of IL-1*α* paracrine effect by a natural antagonist IL-1Ra also resulted in a significant delay in tumor growth. Here we evaluated the homologous hypothesis test and traditional two-sample *t*-test in comparing the tumor volumes between the scrIL-1*α* and IL-1Ra groups across the time points from day 3 to day 24 (n=4 in each group). Table 1 and Figure 2 show that the standard errors of the estimated mean tumor volumes are remarkably smaller than those from the standard method. Correspondingly, the homologous test achieves higher power than the two-sample *t*-test. This makes it possible to detect the difference between two groups at day 21. On the other hand, the *t*-test did not find any significant differences at α=0.05. It is notable that this significant gain in power is attributed to the high correlation between tumor volumes at neighboring time points of measurements (Table 1).

## 6 Conclusions

In this manuscript, we have presented a straightforward approach to harnessing the correlation structure between time-points in a cross-sectional analysis of mean tumor volumes. Our novel method, the homologous hypothesis approach, offers significant advantages in terms of statistical power, especially when faced with a fixed sample size or the need to reduce sample sizes and costs while maintaining a fixed power, as compared to the traditional *t*-test.

One of the key strengths of our method is its simplicity, as it allows for a clear and efficient implementation of the analysis using time moving forward. Nevertheless, we recognize that there are opportunities for further advancements and extensions to our approach.

For instance, future investigations could explore the use of multiple time-points in either direction along the time scale. Incorporating additional time-points could potentially enhance the precision of our results and provide a more comprehensive understanding of the treatment effects over time.

Furthermore, an exciting avenue for future research lies in developing methods to combine p-values across multiple tests for a global assessment of treatment effects over time. This would offer a more holistic perspective on the efficacy of the treatments under investigation and could lead to more robust and insightful conclusions.

In conclusion, our work represents an important step towards a more powerful and flexible approach for analyzing mean tumor volumes in cross-sectional studies. While we have presented the most straightforward version of our method using time moving forward, there is considerable potential for further enhancement and expansion, which could open up new possibilities for the analysis of time-dependent data in medical research. We hope that our findings will inspire further investigations and foster the development of innovative statistical methods in this domain.

## Figures and Tables

**Table 1:** The estimated mean tumor volumes and standard errors (SEs) using the proposed and conventional methods. The Pearson’s correlation coefficients with tumor volume at previous time point ρ and *p*-values from the test of homologous hypothesis and two-sample *t*-tests are also shown.

Time (day)	3	5	7	12	14	17	19	21	24

IL-1Ra									
Tumor volume	4.025	28.725	28.025	36.225	71.725	113.025	131.600	174.125	247.775
SE (Homologous)	0.006	3.456	0.994	1.543	1.905	7.100	7.972	4.736	5.215
SE	0.006	6.731	3.413	7.143	16.305	13.315	17.215	21.086	23.390
ρ	–	–	0.911	0.952	0.986	0.657	0.756	0.948	0.949

scrIL-1*α*									
Tumor volume	4.025	28.150	36.775	41.425	58.675	79.750	102.325	115.925	197.150
SE (Homologous)	0.006	1.042	2.673	5.597	4.256	5.738	8.948	7.915	9.480
SE	0.006	2.806	5.279	7.918	11.198	19.222	25.563	35.908	60.088
ρ	–	–	0.698	0.029	0.843	0.907	0.869	0.950	0.975

*p*-value (Homologous test)	1	0.931	0.151	0.632	0.181	0.103	0.231	0.022	0.054
*p*-value (*t*-test).	1	0.940	0.213	0.643	0.534	0.205	0.379	0.212	0.462

**Table 2:** Simulated power results given σ, $\beta_{1,j}$, $\beta_{2,j}$ and $\beta_{3,j}$ for the homologous and standard cross-sectional hypotheses.

σ	$\beta_{1,j}$	$\beta_{2,j}$	$\beta_{3,j}$	Homologous Power	*t*-Test Power

0.4	0	0	0	0.038	0.051
0.4	0	0	0.5	0.043	0.057
0.4	0	0	1	0.042	0.051

0.4	0	0.5	0	0.043	0.045
0.4	0	0.5	0.5	0.047	0.061
0.4	0	0.5	1	0.042	0.047

0.4	0	1	0	0.054	0.055
0.4	0	1	0.5	0.042	0.053
0.4	0	1	1	0.048	0.043

0.4	0.5	0	0	0.541	0.562
0.4	0.5	0	0.5	0.528	0.403
0.4	0.5	0	1	0.513	0.239

0.4	0.5	0.5	0	0.545	0.302
0.4	0.5	0.5	0.5	0.557	0.226
0.4	0.5	0.5	1	0.533	0.17

0.4	0.5	1	0	0.555	0.155
0.4	0.5	1	0.5	0.56	0.131
0.4	0.5	1	1	0.556	0.13

0.4	1	0	0	0.961	0.975
0.4	1	0	0.5	0.963	0.868
0.4	1	0	1	0.971	0.565

0.4	1	0.5	0	0.963	0.728
0.4	1	0.5	0.5	0.97	0.505
0.4	1	0.5	1	0.954	0.321

0.4	1	1	0	0.968	0.386
0.4	1	1	0.5	0.966	0.299
0.4	1	1	1	0.969	0.242

**Table 3:** Simulated power results given σ, $\beta_{1,j}$, $\beta_{2,j}$ and $\beta_{3,j}$ for the homologous and standard cross-sectional hypotheses.

σ	$\beta_{1,j}$	$\beta_{2,j}$	$\beta_{3,j}$	Homologous Power	*t*-Test Power

0.7	0	0	0	0.051	0.049
0.7	0	0	0.5	0.053	0.054
0.7	0	0	1	0.06	0.057

0.7	0	0.5	0	0.05	0.056
0.7	0	0.5	0.5	0.045	0.045
0.7	0	0.5	1	0.054	0.049

0.7	0	1	0	0.054	0.049
0.7	0	1	0.5	0.038	0.043
0.7	0	1	1	0.049	0.051

0.7	0.5	0	0	0.253	0.265
0.7	0.5	0	0.5	0.265	0.256
0.7	0.5	0	1	0.27	0.179

0.7	0.5	0.5	0	0.267	0.22
0.7	0.5	0.5	0.5	0.262	0.182
0.7	0.5	0.5	1	0.272	0.165

0.7	0.5	1	0	0.29	0.153
0.7	0.5	1	0.5	0.248	0.123
0.7	0.5	1	1	0.286	0.108

0.7	1	0	0	0.63	0.654
0.7	1	0	0.5	0.646	0.589
0.7	1	0	1	0.622	0.413

0.7	1	0.5	0	0.641	0.505
0.7	1	0.5	0.5	0.621	0.402
0.7	1	0.5	1	0.617	0.301

0.7	1	1	0	0.664	0.346
0.7	1	1	0.5	0.623	0.254
0.7	1	1	1	0.632	0.222

**Table 4:** Simulated power results given σ, $\beta_{1,j}$, $\beta_{2,j}$ and $\beta_{3,j}$ for the homologous and standard cross-sectional hypotheses.

σ	$\beta_{1,j}$	$\beta_{2,j}$	$\beta_{3,j}$	Homologous Power	*t*-Test Power

1	0	0	0	0.051	0.047
1	0	0	0.5	0.038	0.045
1	0	0	1	0.057	0.049

1	0	0.5	0	0.038	0.036
1	0	0.5	0.5	0.051	0.051
1	0	0.5	1	0.044	0.06

1	0	1	0	0.056	0.056
1	0	1	0.5	0.061	0.055
1	0	1	1	0.054	0.046

1	0.5	0	0	0.163	0.163
1	0.5	0	0.5	0.174	0.159
1	0.5	0	1	0.179	0.158

1	0.5	0.5	0	0.17	0.165
1	0.5	0.5	0.5	0.164	0.136
1	0.5	0.5	1	0.163	0.103

1	0.5	1	0	0.176	0.126
1	0.5	1	0.5	0.191	0.126
1	0.5	1	1	0.157	0.106

1	1	0	0	0.413	0.429
1	1	0	0.5	0.403	0.383
1	1	0	1	0.409	0.325

1	1	0.5	0	0.401	0.374
1	1	0.5	0.5	0.391	0.311
1	1	0.5	1	0.433	0.27

1	1	1	0	0.424	0.282
1	1	1	0.5	0.398	0.236
1	1	1	1	0.386	0.178

## Data Availability

All data generated or analysed during this study are included in a supplemental file.
